# Enhanced Responsivity, Accuracy, and Stability of Aerosol Jet Printing via Mechanical Switching Valve Assisted Internal Shuttering

**DOI:** 10.1002/advs.202519959

**Published:** 2025-11-29

**Authors:** Jinhang Wang, Li Meng, Shuhuan Zhang, Pan Chen, Kaiwen Wei, Beibei Zhu, Xiaoyan Zeng

**Affiliations:** ^1^ Wuhan National Laboratory for Optoelectronics School of Optical and Electronic Information Huazhong University of Science and Technology Wuhan Hubei 430074 China

**Keywords:** aerosol jet printing, high‐accuracy manufacturing, jet relaxation, ON‐OFF delay, shuttering system

## Abstract

Aerosol jet printing (AJP) is a promising direct writing (DW) technology based on the gas‐driven aerosol. However, the pressure within printhead is hard to be controlled in real time, leading to obvious jet relaxation phenomenon during AJP, which is the critical determinant affecting dimensional accuracy and conformity of printed patterns. In this work, a shuttering system based on an internal mechanical switching valve with special flow channels is proposed to modulate the pressure distribution within printhead, thereby controlling the aerosol jet stream's flow direction in real time to enable faster ON‐OFF responsivity and higher printing accuracy. By designing the flow channel geometry of valve, the pressure is maintained constant during ON‐OFF switching, fundamentally eliminating jet relaxation time from > 35 s, improving morphological uniformity along entire printed lines, permitting parameter‐independent characteristics. With this strategy, the ON‐OFF delay due to the dimensions of the two dead zones in AJP system is eliminated by precompensation. Moreover, the stability and universality of this approach are analyzed by repeatedly printing short‐line arrays at ON‐OFF frequency *F* = 0.2–50 Hz, aligned endpoints demonstrate the stable high‐responsivity ON‐OFF control characteristics, which confirms great application prospects of this strategy in high‐accuracy manufacturing of complex functional patterns.

## Graphical abstract

This work demonstrates ultra‐high ON‐OFF responsivity of AJP by mechanical switching valve assisted shuttering. The jet relaxation and ON‐OFF delay are substantially eliminated from greater than 35 s, maintaining patterning quality independent of the variations of printing parameters, permitting excellent process stability and universality. This leads to significant improvement in manufacturing accuracy of AJP for complex functional devices.

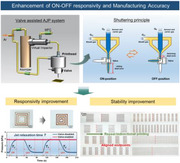

## 1 Introduction

Compared with traditional technologies such as screen printing,^[^ ^1^ ^]^ photolithography and so on,^[^ ^2^ ^]^ DW technology enables the mask‐free manufacturing of patterns directly onto substrates, overcoming the structural limitations inherent in traditional manufacturing processes.^[^ ^3^, ^4^ ^]^ Therefore, it shows great application prospects in manufacturing of 3Dconformal circuits and has garnered significant research interest in recent years.^[^ ^5^, ^6^, ^7^ ^]^ AJP is a non‐contact DW technique based on aerosol,^[^ ^8^, ^9^ ^]^ in which the atomized aerosol droplets are transported to printhead by an inert gas (as carrier gas) and annularly surrounded by another inert gas (as sheath gas) to form collimated aerosol jet stream, enabling the precise deposition of functional patterns onto the substrate. With a stand‐off distance (the gap between nozzle and substrate) of up to 5 mm, AJP technology is granted significant advantages in conformal printing on curved substrates because of the freedom in physical space.^[^ ^10^, ^11^ ^]^ Therefore, the AJP technology has garnered widespread attentions recently and achieved applications in the manufacture of conformal antennas,^[^ ^12^, ^13^, ^14^ ^]^ flexible display screens,^[^ ^15^, ^16^ ^]^ sensors,^[^ ^17^, ^18^, ^19^, ^20^ ^]^ solar cells,^[^ ^21^, ^22^ ^]^ and other functional devices.^[^ ^23^, ^24^ ^]^

Ramesh et al. revealed the effects of gas flow rates, nozzle shape, stand‐off distance, etc., on the accuracy and morphologies of printed features by establishing a comprehensive computational fluid dynamics (CFD) framework.^[^ ^25^ ^]^ Mosa et al. proposed a laser scattering technique to visualize the focused spray jet to determine both the aerosol beam's jet diameter and its breakdown length, and 3D printing with a stand‐off distance exceeding 10 mm on a complex surface was successfully achieved.^[^ ^26^ ^]^ Ma et al.c introduced an annular acoustic focusing field outside the AJP nozzle, by which ultrafine traces with line width < 6 µm and overspray < 0.1 µm were obtained, improving the printing accuracy of AJP technology greatly.^[^ ^27^ ^]^ Li et al. fabricated an optically transparent printed planar inverted‐F antenna (PIFA) by AJP, which exhibited a wide fractional bandwidth (FBW) of 20% centered at 2.45 GHz, with a peak realized gain of −3.6 dBi and transparency of ≈80%.^[^ ^13^ ^]^ Ritchie et al. achieved the support‐free manufacturing of 3D microstructures including spirals, microcolumns, microfilaments and micro‐lattices by AJP, which had great application prospects in implantable biosensors, microelectronic circuits and catalytic devices.^[^ ^28^, ^29^, ^30^ ^]^ Zeng et al. demonstrated a variety of high‐throughput printing strategies and applications of multi‐component functional material, which enabled the manufacture of compositionally graded materials with gradient properties.^[^ ^31^ ^]^

In summary, the current relevant researches on AJP technology mainly focused on how to manufacture patterns with narrow line‐width and little overspray by structure design,^[^ ^27^, ^32^, ^33^ ^]^ model simulation,^[^ ^25^, ^34^ ^]^ and process parameter optimization.^[^ ^35^, ^36^, ^37^, ^38^, ^39^ ^]^ Nonetheless, the conformity of printed patterns to the designed models is also a key factor affecting the deposition quality of AJP technology, which means to print the designed patterns accurately without deviation. During AJP process, aerosol particles are mainly transported through gas flowing, and the gas pressure is the key factor affecting the deposition mass. However, When the ON/OFF control command is carried out, the pressure in the flow channel of printhead will inevitably have a gradual increase/decrease process, resulting in a finite stabilization time required for the aerosol jet stream flow rate to reach a steady state. Namely, there is a jet relaxation time *T* in the AJP ON‐OFF switching, which seriously impacts the conformity of printed patterns. The jet relaxation time *T* includes the ON‐relaxation time *T*_1_ and OFF‐relaxation time *T*_2_, respectively, where the former is defined as the time that takes from the printing command transmission to the stabilization of aerosol jet stream, and the latter is defined as the time that takes from the printing stop command to the time that aerosol jet stream is completely cut off.

In order to solve above issues, Optomec Inc. proposed an external shuttering scheme, in which the aerosol jet stream was blocked through an external mechanical shuttering system beside the nozzle to achieve fast stop of AJP.^[^ ^40^ ^]^ As a result, jet relaxation time *T* was eliminated because the pressure in printhead was maintained constant during printing process, whereas an ON‐OFF jet delay time *t* with value of larger than 2 ms was caused due to the signal response delay and motion inertia of the mechanical shuttering system. However, the external shuttering system has a considerable size, resulting in that the stand‐off distance had to be increased to deviate from the optimal value, which significantly affected the forming quality and applications of patterning on curved substrates. Meanwhile, the ink accumulated on the external shutter surface was prone to redeposit onto the substrate when the printing was resumed, which compromised the integrity of pre‐existing patterns.

To overcome the limitations of the external shuttering mechanisms, a variety of internal shuttering mechanisms was proposed. IDS Inc. proposed an internal pneumatic shuttering scheme,^[^ ^41^ ^]^ in which the carrier gas was stopped and transferred into the sheath gas flow channel by a three‐way valve when printing was stopped, the backpressure of the aerosol stream was increased to achieve its shuttering. Optomec Inc. introduced an additional pressurized gas flow to increase the pressure of sheath gas flow when printing was stopped, so as to realize efficient shuttering by reversing the motion of aerosol stream.^[^ ^42^, ^43^ ^]^ However, there are some constraints in above pneumatic shuttering schemes of AJP. On the one hand, only the aerosol stream that was not surrounded by sheath gas could be blocked and a part of the sheath gas flow was still sprayed from nozzle when printing was stopped, affecting the pre‐printed patterns. On the other hand, it took a while for the pressure in printhead to stabilize during the OFF to ON switching process, resulting in an ON‐relaxation time *T_1_*. Kwon et al proposed an internal mechanical rotary valve assisted AJP system,^[^ ^44^ ^]^ in which fast ON to OFF control of aerosol stream was achieved by rotating the valve to switch its flow path. Unfortunately, there still existed an ON‐relaxation time *T*_1_ of ≈18 s due to the pressure variation in printhead during the OFF to ON switching process. By adjusting the exhaust gas flow rate *Q_E_* in real time, the pressure in printhead was restored as quickly as possible, and the ON‐relaxation time *T*_1_ was shortened to ≈4 s. However, the optimal *Q*_E_ needed to be re‐investigated in‐depth as the AJP parameters were adjusted, including the carrier gas flow rate, sheath gas flow rate and so on, making the scheme was hard to put into practice in applications. In addressing above problems, Kwon et al. proposed an upgraded pneumatic shuttering scheme of AJP system,^[^ ^45^ ^]^ a boost gas was combined directly with sheath gas, constant pressure during ON‐OFF switching of AJP was achieved by switching the direction of boost gas with a solenoid valve and a vent MFC. Unfortunately, there was still sheath gas flow outside the nozzle to impact the printed pattern during shuttering, and the mist flow needed to re‐enter the printhead to be focused during printing, resulting in a slight jet delay.

In this work, a novel AJP system with high ON‐OFF responsivity (i.e., no jet relaxation and ON‐OFF delay) was proposed by introducing an internal switching valve as shutter with optimized flow channel geometry, whereby the aerosol jet stream surrounded by sheath gas could be cut off timely when printing was stopped so as the adverse effect of gas flow impact on the pre‐printed patterns could be avoided, and the pressure within printhead during ON‐OFF switching process could be remained constant so as the jet relaxation time *T* could be eliminated. First, the pressure distribution characteristics within printhead and the corresponding topography characteristics of printed lines under different inner diameters of the valve OFF‐channel were analyzed, by which the optimal valve flow channel geometry was determined. Second, the jet relaxation characteristics of the AJP system were investigated systematically under varying printing parameters. Next, the ON‐OFF delay time *t* of the AJP system were revealed and the compensation strategies were proposed. Accordingly, the ON‐OFF switching stability of the optimized AJP system under different switching frequencies *F* and the generalization for other inks formulations were demonstrated. Based on the above, a series of functional devices with complex patterns were manufactured by the AJP system to demonstrate its feasibility in large‐format patterning. To the best of our knowledge, this shuttering scheme of AJP system demonstrated the shortest jet relaxation and ON‐OFF delay.

## 2 Results and Discussion

**Figure 1:**
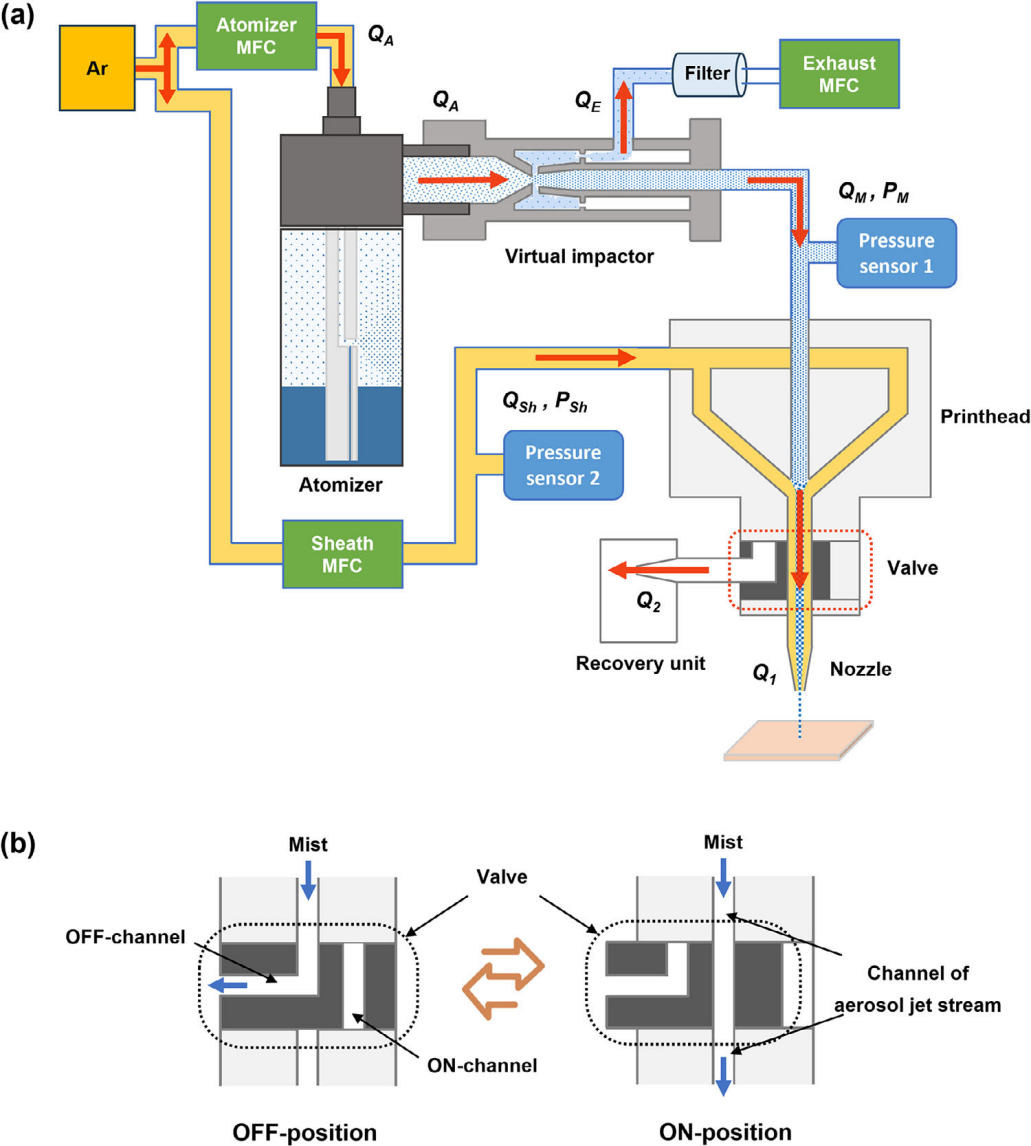
Schematic diagrams showing the a) whole AJP system with an internal mechanical switching valve and b) working mechanism of the switching valve.

To improve the ON‐OFF responsivity of AJP for higher manufacturing accuracy, a self‐made AJP system assisted by an internal mechanical switching valve with special flow channels (abbreviated as switching valve below) was developed in this paper, aiming at demonstrating a novel internal pressure‐balancing regulation mechanism for the AJP system during ON‐OFF switching process. As shown in **Figure** **1**, there are two independent flow channels in the switching valve: ON‐channel and OFF‐channel. The inlet direction of ON‐channel and OFF‐channel are identical, while the outlets are positioned beneath and alongside the valve respectively. When the printing is initiated, the ON‐channel of valve is switched to the path of the aerosol jet stream channel, and the jet stream can be deposited on substrate after passing through the nozzle along the ON‐channel, the flow rate at nozzle *Q*_1_ = *Q*_M_+*Q*_Sh_. When the printing is stopped, the OFF‐channel of valve is switched to the path of the aerosol jet stream channel, whereby the jet stream flows out of the printhead along the OFF‐channel and into the recovery unit. At this time, the flow rate at nozzle *Q*_1_ = 0 and the flow rate at the exit of OFF‐channel *Q*_2_ = *Q*_M_+*Q*_Sh_. The photograph of the AJP system is shown in Figure S2 (Supporting Information).

### 2.1 Effect Characteristics of Valve Structure on Internal Pressure

In AJP process, the atomized aerosol particles were carried by gas to deposit on substrates, and the flow rate of aerosol jet stream outside the nozzle was mainly determined by the pressure in printhead, which had a direct effect on the deposition quality of patterns. In our design scheme, the aerosol jet stream would be completely directed away through the elbow structure of the switching valve during shuttering. As shown in Figure 1, a 90° elbow was designed in the OFF‐channel of the valve. According to the pressure loss theory for elbow,^[^ ^46^ ^]^ the change in flow direction causes part of the fluid's mechanical energy to be dissipated, thereby altering the pressure distribution inside the printhead. The local pressure loss of the elbow can be calculated using the resistance coefficient method (Equation 1):

(1) $$\Delta P=\zeta \cdot \frac{1}{2}\rho v^{2}$$

where Δ*P* is the pressure loss, *ζ* is the local resistance coefficient of the elbow, *ρ* is the fluid density, and *v* is the average flow velocity of fluid.

The local resistance coefficient *ζ* is mainly influenced by multiple parameters, including the bend angle, channel diameter, and fluid Reynolds number. To quantitatively determine the pressure loss of aerosol jet stream caused by the elbow and its impact on the pressure distribution inside the printhead, numerical simulations based on the CFD simulation software Fluent was conducted to analyze the complex flow field (velocity, pressure) within the printhead. Specially, to achieve identical internal pressure distribution in printhead during both the printing state and shuttering state, the effect of OFF‐channel's internal diameter in switching valve on the distribution characteristics of pressure in printhead during ON‐OFF switching process was investigated systematically, and the optimized design was obtained. Wherein, the geometry of the ON‐channel was configured to be the same as that of the aerosol jet stream channel.

**Figure 2:**
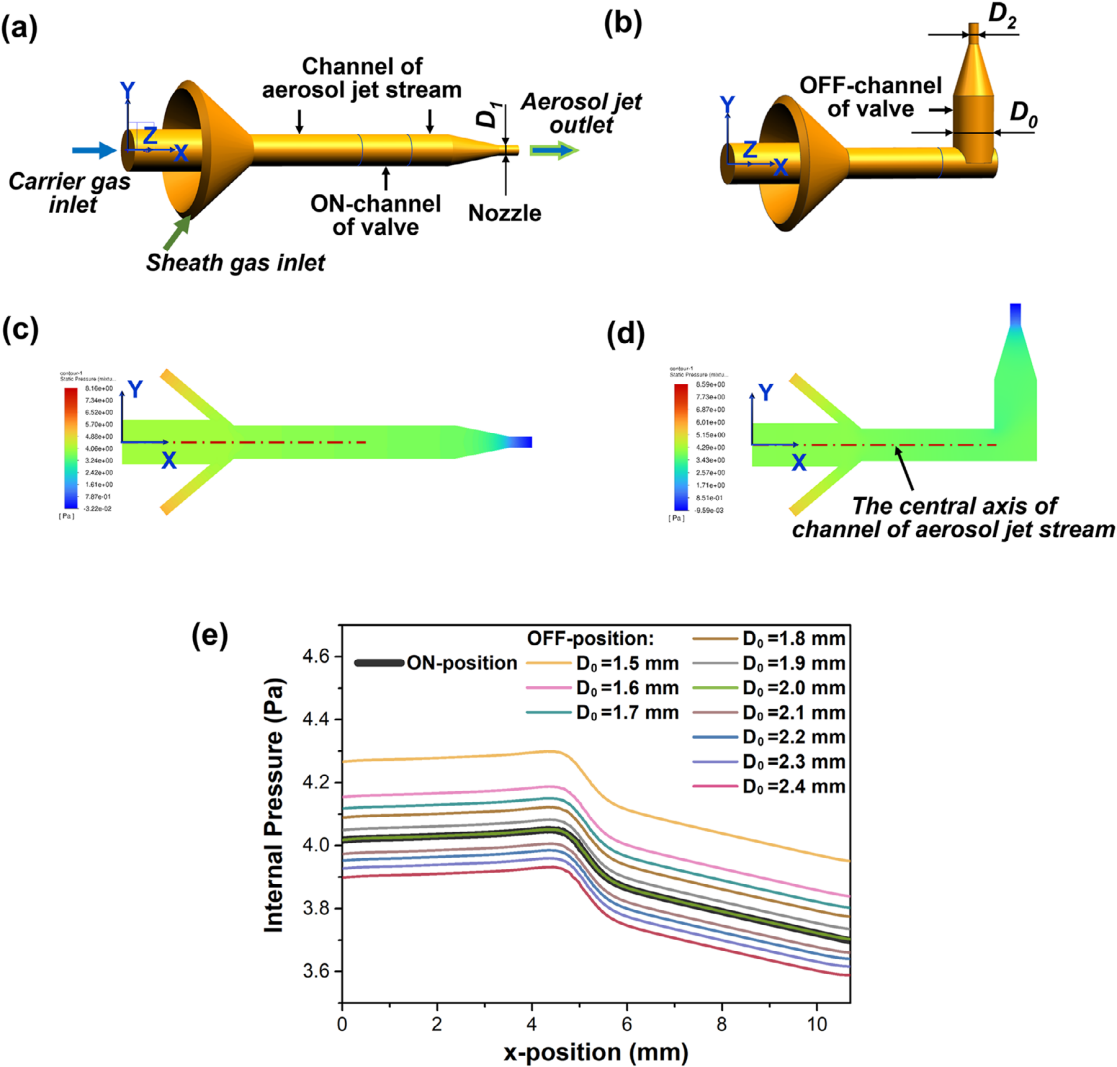
The simulation models of the flow channel within printhead in a) ON‐position, and b) OFF‐position. The pressure distribution nephograms on the cross‐section of *Z* = 0 in c) ON‐position, and d) OFF‐position. e) The pressure distribution curves on the central axis of internal channel within printhead under different *D*_0_.

Based on the schematic diagram of the AJP system shown in Figure 1, the CFD simulation models of the flow channel in printhead were constructed by NX 12.0 software, including that at the valve‐ON and valve‐OFF states, as shown in **Figure** **2**a,b, respectively. The fluid domain models included the carrier gas inlet, sheath gas inlet, mixing chamber, channel of aerosol jet stream, nozzle, and the ON and OFF channels of the switching valve. Four boundary conditions were defined: carrier gas velocity inlet (Inlet 1), sheath gas velocity inlet (Inlet 2), wall (Wall), and pressure outlet (Outlet). In the CFD simulation, the density‐based Navier–Stokes equation was used to solve the multiphase flow problem, and the corresponding pressure distribution nephograms were obtained, as shown in Figure 2c,d.

The pressure distribution characteristics along the central axis of flow channel within printhead under different middle inner diameter *D_0_* of the OFF‐channel were analyzed, in which the velocities of carrier gas and sheath gas were set to 0.1 and 0.5 m s^−1^, respectively, and the outlet inner diameter of the OFF‐channel *D_2_* was set to be same as that of the nozzle *D*_1_, which was 0.5 mm in this work. As shown in Figure 2e, the pressure in printhead progressively decreased with the increase of *D_0_* at valve‐OFF state. Crucially, when *D_0_* = 2.0 mm, the pressure distribution characteristic exhibited identical profiles to that at the valve‐ON state.

**Figure 3:**
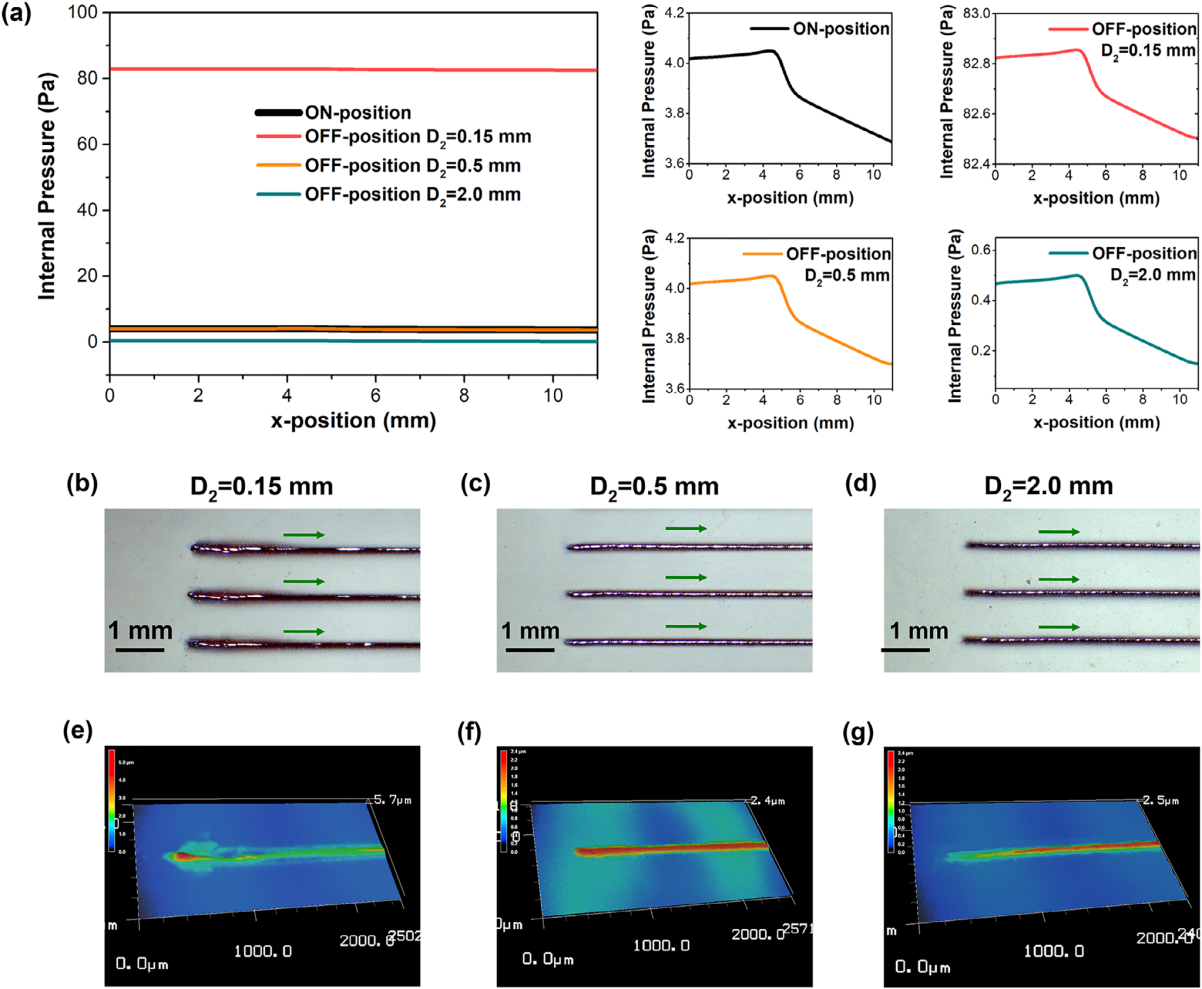
a) The pressure distribution curves on the central axis of internal channel in printhead. b–g) The surface morphologies and 3D topographies of the printed lines head with *D*_2_ of 0.15, 0.5, and 2.0 mm.

Then, the pressure distribution characteristic within the inner channel of printhead under different *D*_2_ were analyzed. In which, *D*_0_ and *D*_1_ were set as 2 mm and 0.5 mm respectively, and the flow rates of carrier gas *Q*_A_ and sheath gas *Q*_Sh_ were set to 70 and 120 sccm, respectively. **Figure** **3**a shows the pressure distribution curves along the central axis of internal channel in printhead at *D*_2_ = 0.15, 0.5, and 2.0 mm, and Figure 3b–g show the corresponding morphologies of the printed lines head. It is evident that the pressure in printhead varied with the variations in *D*_2_, thereby affecting the morphologies of printed lines. When *D*_2_ = 0.15 mm (less than *D*_1_), the internal pressure in printhead at the valve‐OFF state was around 20 times higher than that at the valve‐ON state. In the meantime, the head of the printed lines was piled up, where the width and thickness were significantly greater than those of other parts, as shown in Figure 3a,b,e. When *D*_2_ = *D*_1_ = 0.5 mm, the internal pressure in printhead at the valve‐OFF state was the same as that at the valve‐ON state, and the whole lines displayed the uniform morphologies, as shown in Figure 3a,c,f. When *D*_2_ = 2.0 mm (greater than *D*_1_), the internal pressure in printhead at the valve‐OFF state was reduced to less than around one‐eight of that at the valve‐ON state, while the ink at the head of lines was slightly insufficient, resulting in a shrinking thickness, as shown in Figure 3a,d,g.

In total, when *D*_0_ = 2 mm and *D*_2_ = 0.5 mm, the pressure in printhead could be remained basically stable during the valve ON‐OFF switching cycles, without observable jet relaxation in the AJP process. Consequently, the printed lines exhibited uniform morphologies, and their thickness and width were consistent along the entire length.

### 2.2 Investigation on the ON‐OFF Responsivity of AJP

**Figure 4:**
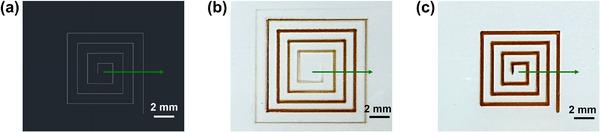
a) Designed pattern. Printed patterns by AJP with b) valve‐disabling and c) valve‐enabling.

On the basis of Section 2.1, an internal mechanical switching valve assisted AJP system with high ON‐OFF responsivity was designed. **Figure** **4** shows the designed and printed spiral patterns by the AJP system, where the green arrows indicate the moving direction of printing path. Under valve‐disabled conditions (the valve has always been in the ON‐position), as shown in Figure 4b, gradual transitions were observed at the head and tail of the printed pattern, demonstrating the evident ON‐relaxation and OFF‐relaxation phenomena. Conversely, with the valve enabled, as shown in Figure 4c, the entire pattern showed uniform morphology, indicating that the ON‐relaxation and OFF‐relaxation in AJP were significantly eliminated, and the printing accuracy was greatly improved.

**Figure 5:**
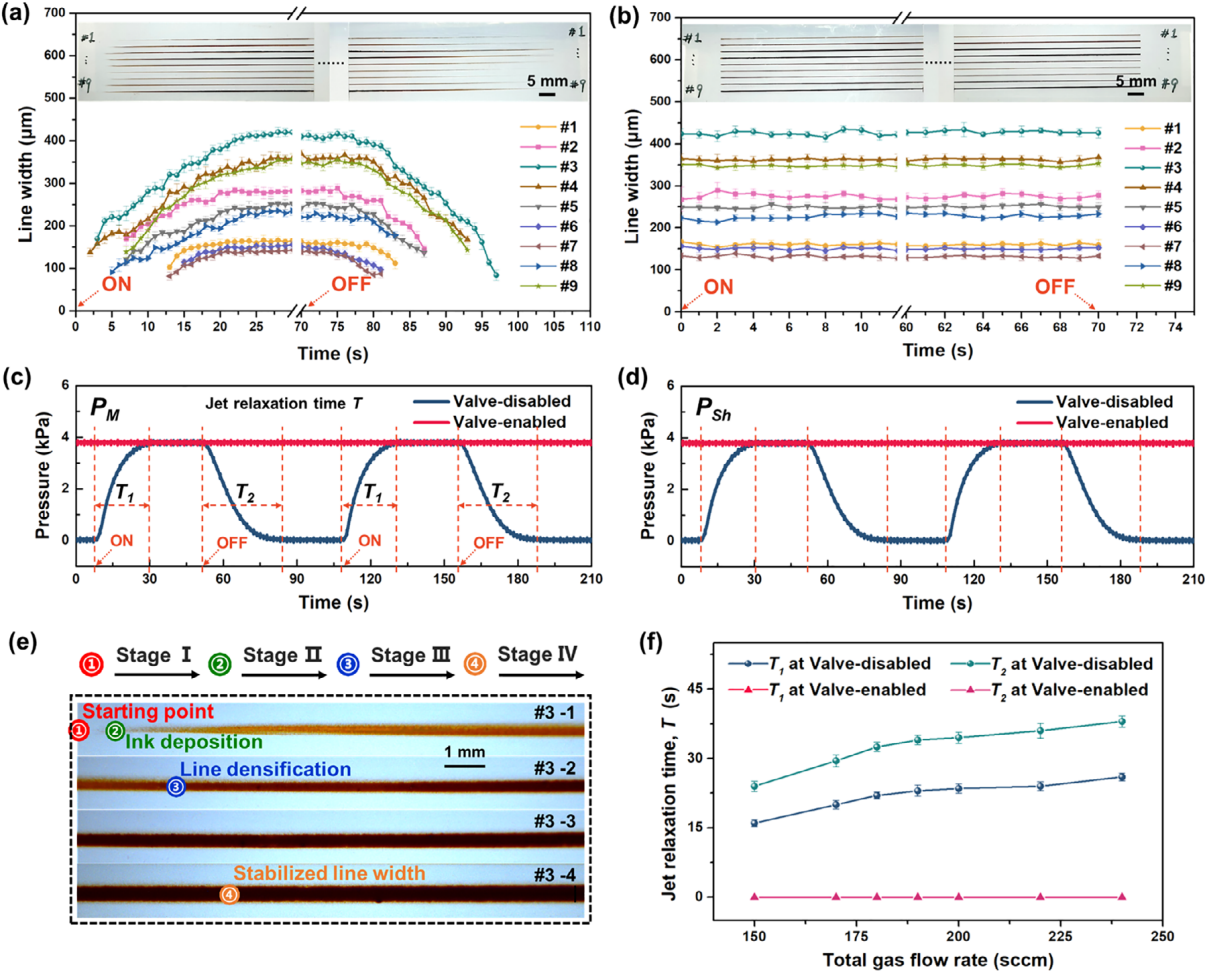
Line width variations under AJP systems with a) valve‐disabling and b) valve‐enabling, the insets show the photographs of the heads and tails of printed lines. Measured internal pressure at c) mist flow tube and d) sheath gas flow inlet in printing process of #3 line. e) Enlarged morphology of #3 line in figure (a). f) *T* dependence of total gas flow rate.

**Figure** **5** shows the morphologies and width of lines printed by AJP under different gas flow rates, and the corresponding gas flow rate parameters are listed in **Table** **1**. According to Figure 5a, when the switching valve was disabled, there were obvious ON‐relaxation zones and OFF‐relaxation zones at the heads and tails of lines under all gas flow rates. Figure 5c,d shows the measured internal pressure at mist flow tube and sheath gas flow inlet during printing of #3 line, the jet relaxation time *T* (including ON‐relaxation time *T*_1_ and OFF‐relaxation time *T*_2_) were evident when printing was initiated and stopped, which induced the gradient line width of the printed lines. Combined with the enlarged morphology of #3 line shown in Figure 5e, the ON‐relaxation process during traditional valve‐disabled AJP could be divided into the following stages:

1. Stage I: When the printing was initiated (*Starting point*), the carrier gas and sheath gas was delivered into the inner flow channel of printhead. However, the internal pressure was insufficient to drive flow toward the nozzle, resulting in that negligible aerosol was deposited on substrate.
2. Stage II: As the internal pressure within printhead gradually increased, the aerosol particles began to accumulate on the substrate (*Ink deposition*). However, the sheath gas flow failed to effectively confine the aerosol stream into a collimated jet, making the depositions show a scattered spot‐like characteristic.
3. Stage III: The focused aerosol jet was formed under the constraint of sheath gas flow, during which the deposition width became progressively narrowed and the lines developed to more stable and denser (*Line densification*). The measurement of line width in Figure 5 began at this position.
4. Stage IV: The pressure in printhead gradually increased until it reached a stable state, and the jet flow rate outside nozzle was also stabilized synchronously, while the line width evolved to a constant value (*Stabilized line width*).

**Table 1:** Gas flow rate parameters of #1–#9 lines in Figure 5a,b.

	#1	#2	#3	#4	#5	#6	#7	#8	#9
Q_A_ [sccm]	80	100	120	120	100	80	80	100	120
Q_sh_ [sccm]	70	70	70	100	100	100	120	120	120

Similarly, when the AJP printing stop command was implemented, above IV‐III‐II‐I stages occurred sequentially until the jet flow rate outside the nozzle dropped to 0 sccm and the ink deposited on substrate disappeared completely.

In contrast, Figure 5b shows the line width obtained by the valve‐enabled AJP system, and the inset photographs illustrate the heads and tails of the lines. It is noticed that the line widths under varying *Q*_A_ and *Q*_sh_ were comparable to that at steady states when the valve was disabled. However, the difference is that when the printing was initiated (switched from valve‐OFF to valve‐ON state), the lines appeared instantly, and their width remained basically constant over time. When the printing was stopped (switched from valve‐ON to valve‐OFF state), the lines disappeared immediately without trailing. As shown in Figure 5c,d, the results indicate that the internal pressure in printhead of the AJP system maintained consistently stable over the ON‐OFF switching process, resulting in exceptional dimensional accuracy in printed patterns. Figure 5f shows the variation curves of the *T* (*T*_1_ and *T*_2_) with the total gas flow rate (*Q*_A_+*Q*_Sh_) during AJP. When the switching valve in AJP was disabled, the *T* was greater than 35 s and tended to increase as the total gas flow rate increasing. Conversely, under valve‐enabled condition, the *T*_1_ showed negligible values with no dependence on total gas flow rate, validating the excellent elimination effect of jet relaxation.

### 2.3 Analysis of the ON‐OFF Delay

**Figure 6:**
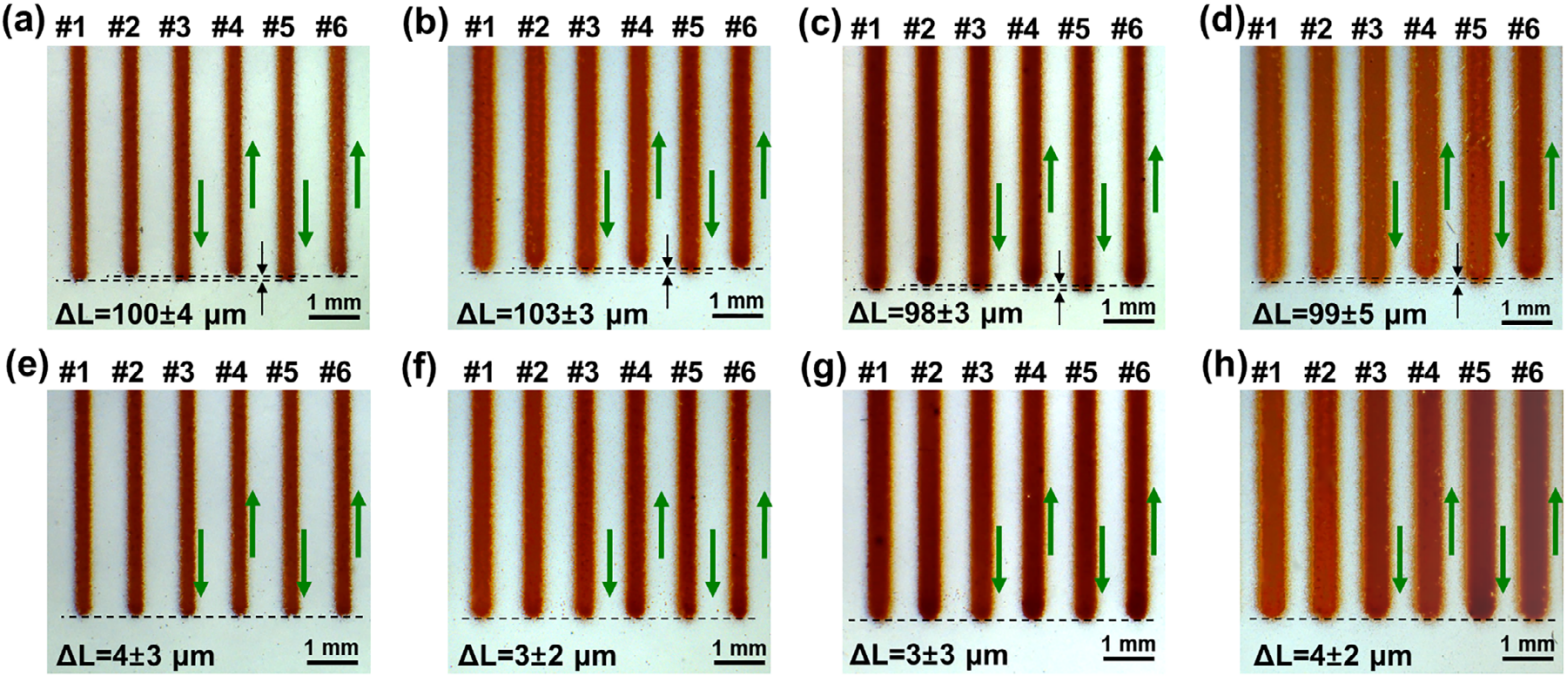
Patterns of printed lines under different gas flow rates a–d) before and e–h) after displacement compensation.

In order to demonstrate the ON‐OFF control responsivity of aerosol jet stream during AJP, a number of parallel lines were printed in bidirectional mode. Compared with unidirectional printing in Section 2.2, bidirectional printing not only allows evaluation of the repeatability and stability of ON‐OFF control by observing the heads and tails of same‐direction lines, but also enables analysis of the difference between ON‐delay and OFF‐delay by examining the overlap consistency at the heads and tails of opposite‐direction lines. **Figure** **6**a–d presents the head/tail morphologies of parallel lines printed bidirectionally under different gas flow rates, with each line labeled with its printing direction, and the corresponding printing parameters are listed in **Table** **2**. It can be observed that none of the lines exhibited gradual transition zones at their head/tail, demonstrating the absence of jet relaxation phenomenon during ON‐OFF switching. The head/tail of same‐direction printed lines showed good alignment (e.g., #1 and #3; #2 and #4), whereas a consistent distance Δ*L* of ≈100 µm existed between the endpoints of opposite‐direction printed lines (e.g., #1 and #2, #3 and #4). The Δ*L* remained essentially unchanged across different gas flow rates, corresponding to an ON‐OFF delay time *t* of 50 ms.

**Table 2:** Gas flow rate parameters of lines in Figure 6(a–h).

	(a,e)	(b,f)	(c,g)	(d,h)
Q_A_ [sccm]	100	100	110	110
Q_sh_ [sccm]	70	60	70	60

**Figure 7:**
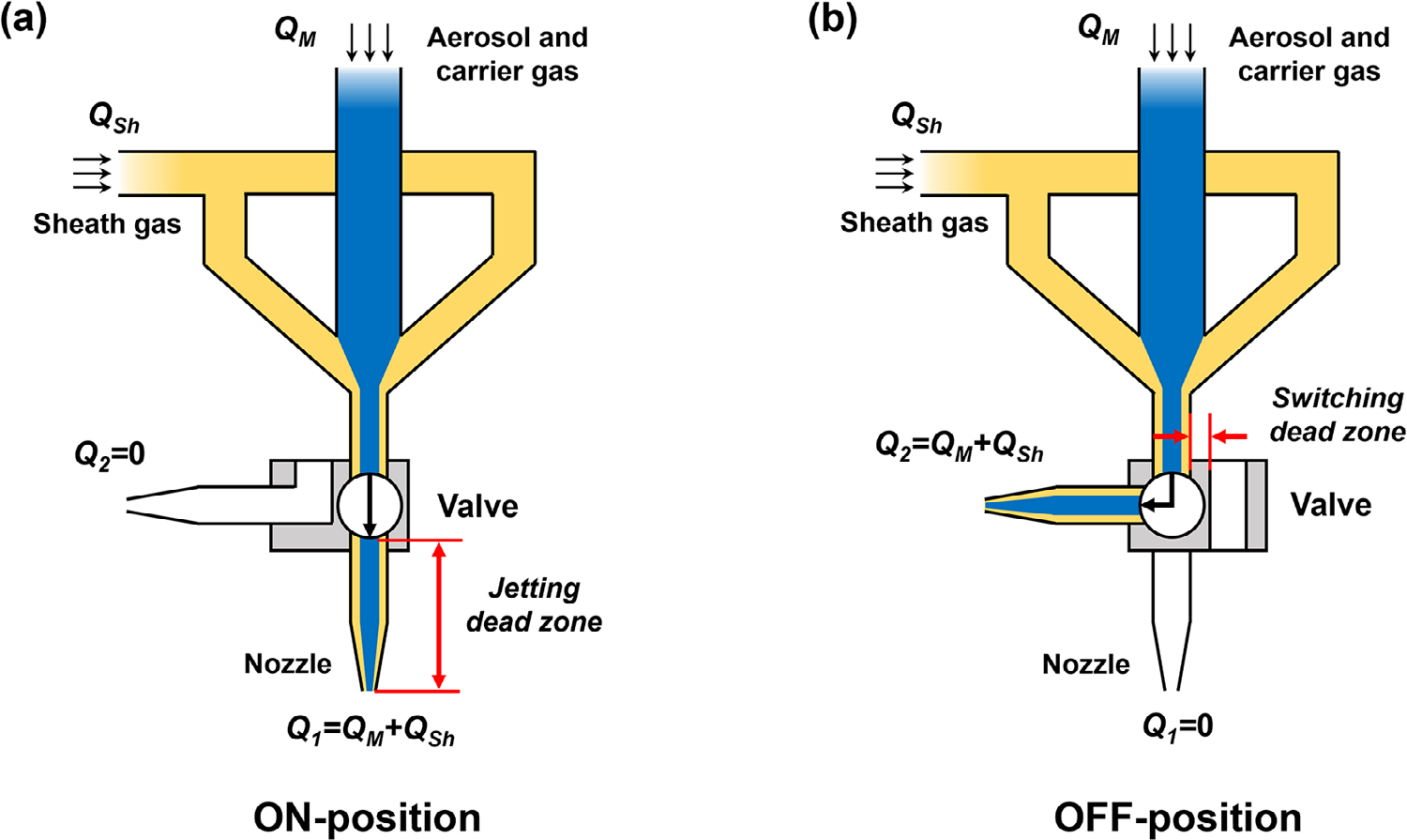
Schematics showing the flow path of aerosol and sheath gas at a) valve‐ON state and b) valve‐OFF state.

Then, the primary cause of the Δ*L* was identified. As illustrated in **Figure** **7**, there exist a jetting dead zone between the switching valve and nozzle, along with a switch dead zone between two channels of the valve. When switching the aerosol jet from the valve's ON‐channel to OFF‐channel to terminate printing, the residual aerosol within the jetting dead zone maintained continuous deposition on substrate, resulting in an OFF‐delay of AJP. Conversely, when the printing was restarted, the aerosol needed to fill the jetting dead zone before deposition commenced, resulting in an ON‐delay of AJP. Combined with the jet velocity calculated by the CFD numerical method in Section 2.1, the ON‐OFF delay time contributed by the jetting dead zone was ≈5 ms. Additionally, during the ON‐OFF switching process, the aerosol needed to pass through the switch dead zone when transitioning from the valve's OFF‐channel to the ON‐channel, introducing a measurable ON‐OFF delay time of ≈45 ms, which could be greatly reduced by increasing the traction force of the control system. In total, above two factors caused the total ON‐OFF delay of 50 ms, corresponding to the accuracy error Δ*L* of 100 µm between the endpoints of opposite‐direction printed lines. In this work, the ON‐OFF delay was overcome by the displacement compensation, in which Δ*L* was incorporated into the original design patterns for printing. As shown in Figure 7e–h, the Δ*L* in bidirectionally printed lines had been eliminated, confirming the effectiveness compensation of the ON‐OFF delay.

### 2.4 Investigation on the ON‐OFF Switching Stability and Generalization for Other Inks Formulations

On the basis of optimized flow channel structure and ON‐OFF delay compensation, the switching stability of AJP system under different ON‐OFF frequency and its compatibility with inks of different viscosities were further investigated in this section, which is crucial for the large‐format high‐accuracy printing and wide application.

**Figure 8:**
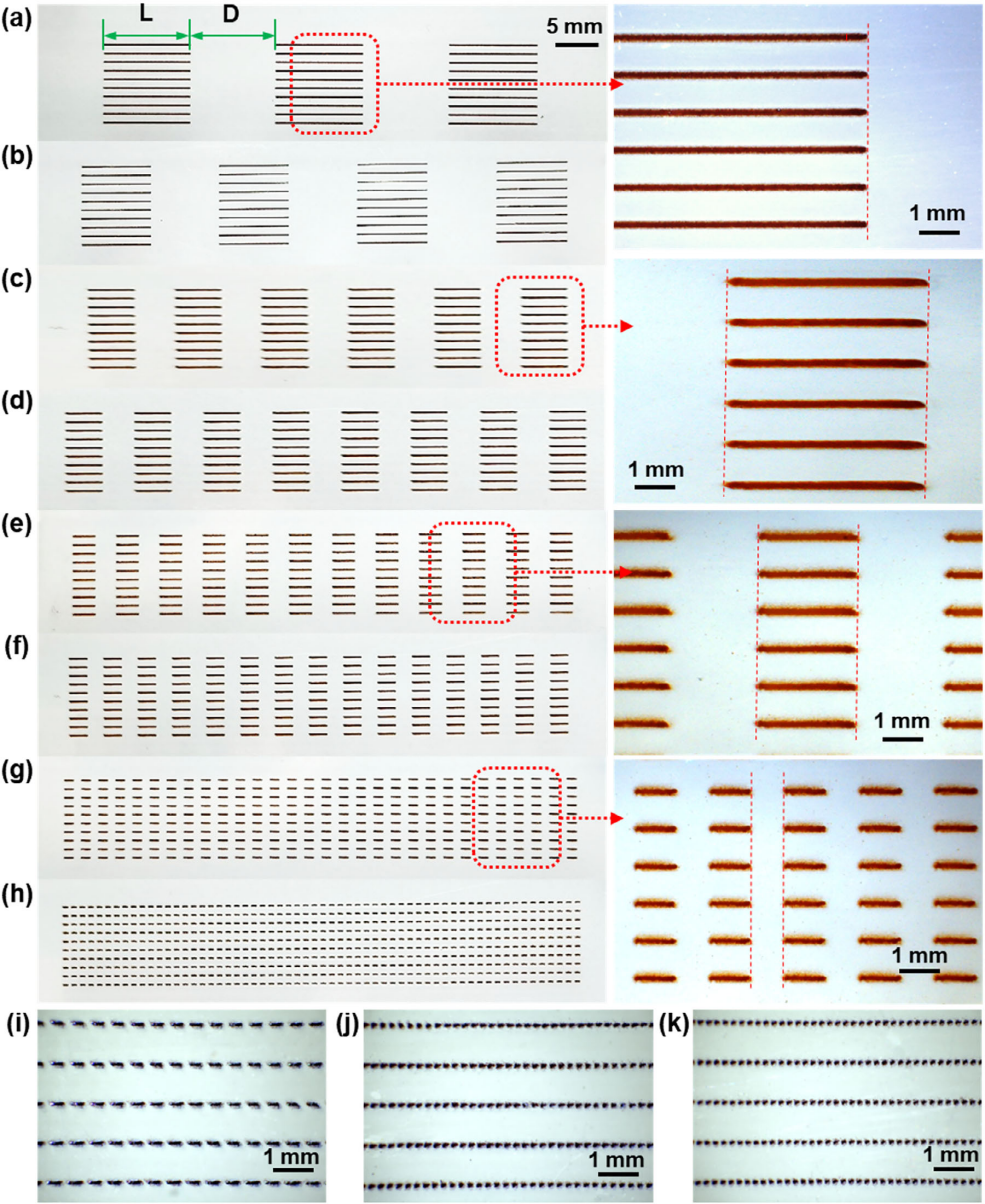
Overall and locally‐magnified images of short‐line arrays printed with a nozzle of a–h) *D*_1_ = 500 µm under ON‐OFF switching frequencies of 0.2–10 Hz, and i–k) *D*_1_ = 200 µm under ON‐OFF switching frequencies of 20–50 Hz.

Eight‐group spatially segmented short‐line arrays were printed by changing the ON‐OFF frequency, with each group comprising ten replicated parallel line arrays. Detailed parameters are provided in **Table** **3**, with stage movement speeds in the ON and OFF states of AJP were fixed at 2 and 5 mm s^−1^. respectively, while the *Q*_A_ and *Q*_sh_ were set to 80 and 100 sccm. A nozzle with 500 µm was used at ON‐OFF switching frequency *F* = 0.2–10 Hz (the line lengths ranging from 10 to 0.5 mm), while a smaller nozzle (200 µm) was employed at higher switching frequencies *F* = 20–50 Hz (the line lengths ranging from 0.25 to 0.1 mm) to minimize the impact of line width on the resolution limit. The jet relaxation elimination under different ON‐OFF switching frequencies was evaluated by examining the morphological consistency at the heads and tails of lines, and the switching stability was assessed through endpoint alignment analysis in the short‐line arrays. As shown in **Figure** **8**, there was no observable gradual transitions in the printed lines with switching frequencies ranging from 0.2 to 50 Hz, and all lines showed good alignment without detectable Δ*L*, which indicated the absolutely elimination of the jet relaxation and ON‐OFF delay, demonstrating that the AJP system had maintained the accurate jet control as ON‐OFF frequency increasing to 50 Hz, and it showed stable transient response.

**Table 3:** Detail printing parameters of the short‐line arrays in Figure 8(a–k).

No.	L = D [mm]	ON‐OFF frequency [Hz]	OFF‐ON frequency [Hz]	Diameter of nozzle, [µm]
(a)	10	0.2	0.5	500
(b)	8	0.25	0.625	
(c)	5	0.4	1	
(d)	4	0.5	1.25	
(e)	2.5	0.8	2	
(f)	2	1	2.5	
(g)	1	2	5	
(h)	0.5	4	10	
(i)	0.25	8	20	200
(j)	0.125	16	40	
(k)	0.1	20	50	

**Figure 9:**
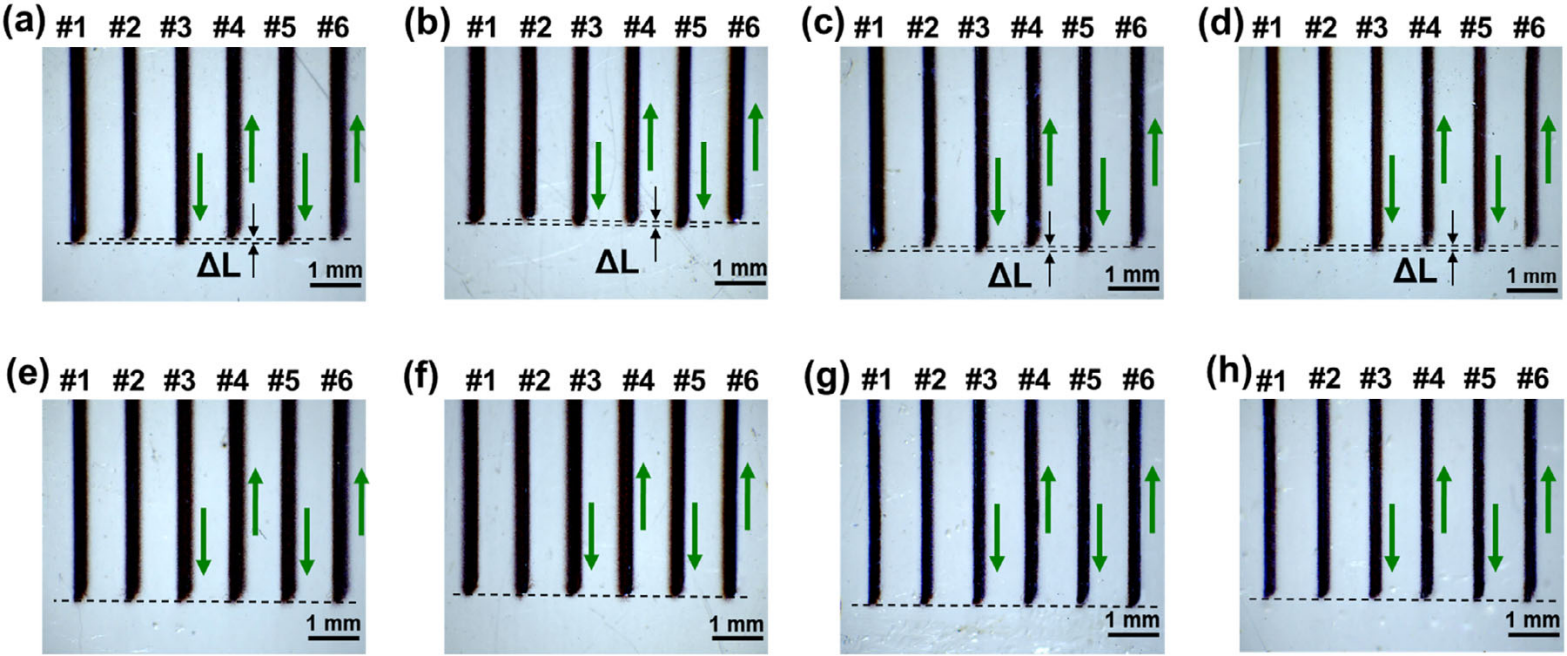
Line patterns printed with nano‐silver ink of viscosity 30 cp a–d) before and e–h) after displacement compensation.

**Figure 10:**
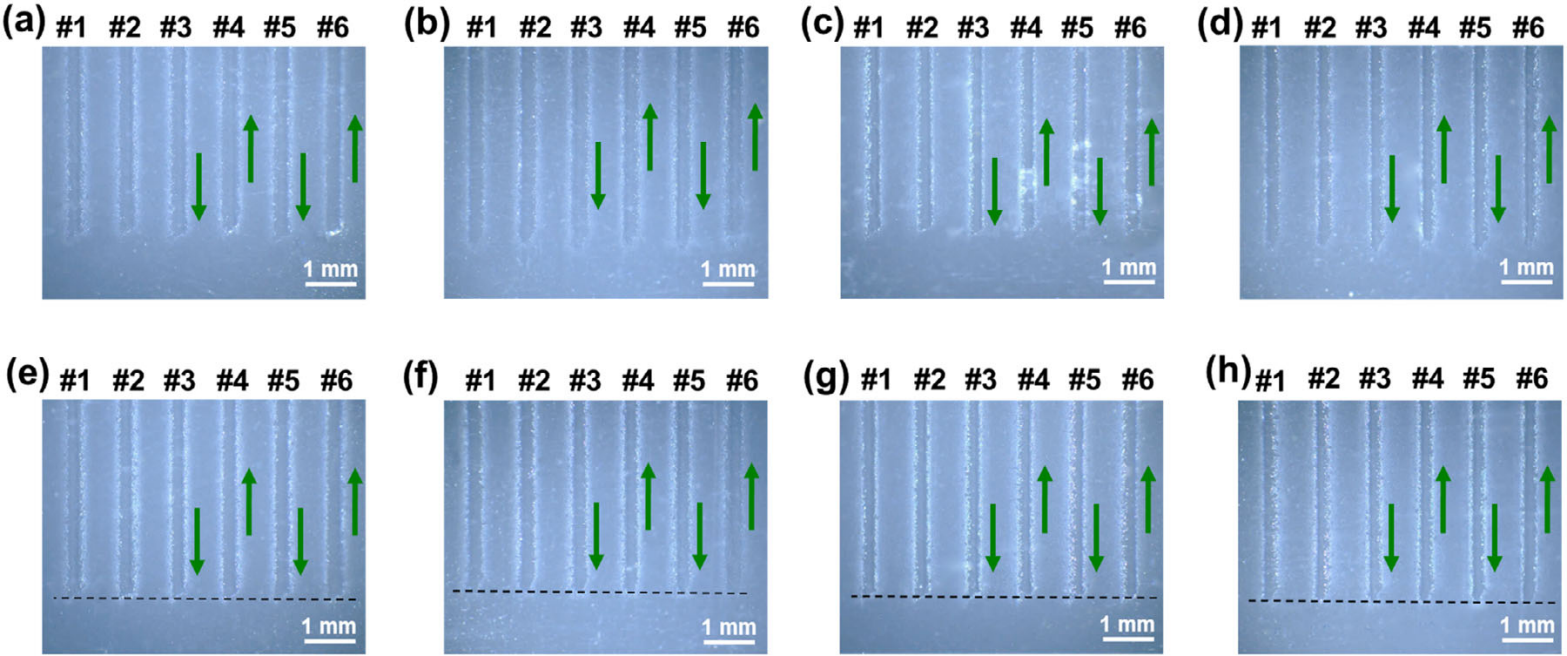
Line patterns printed with particle‐free silver ink of viscosity 117 cp a–d) before and e–h) after displacement compensation.

Further, additional inks with disparate viscosities were printed to evaluate the wide applicability of AJP system proposed in our paper, including an ink with higher solids content (Viscosity: 30 cp) and a particle‐free solution‐based silver ink (Viscosity: 117 cp). **Figures** **9** and **10** show the head/tail morphologies of parallel lines, and the corresponding printing parameters are listed in **Tables** **4** and **5**. The results demonstrated that a higher gas flow rate was required to atomize higher‐viscosity materials. Nonetheless, the jet relaxation was eliminated by the shuttering scheme of our AJP system. In all cases, the Δ*L* remained ≈100 µm, with a corresponding ON‐OFF delay time *t* of 50 ms, which further confirmed that the *t* is primarily determined by the dimensions of two dead zones and the movement speed of the valve control system, independent of the ink properties. This ON‐OFF delay could also be eliminated using the compensation scheme proposed in Section 2.3. These findings validated the versatility of our shuttering scheme across different material types, which indicated that it could be extended to the printing of other electronic ink, dielectric ink as well as structural functional ink.

**Table 4:** Gas flow rate parameters of lines in Figure 9(a–h).

	(a,e)	(b,f)	(c,g)	(d,h)
Q_A_ [sccm]	80	80	80	80
Q_sh_ [sccm]	80	90	100	110

**Table 5:** Gas flow rate parameters of lines in Figure 10(a–h).

	(a,e)	(b,f)	(c,g)	(d,h)
Q_A_ [sccm]	150	150	150	150
Q_sh_ [sccm]	100	110	120	130

### 2.5 Printing of Complex Patterns by AJP

**Figure 11:**
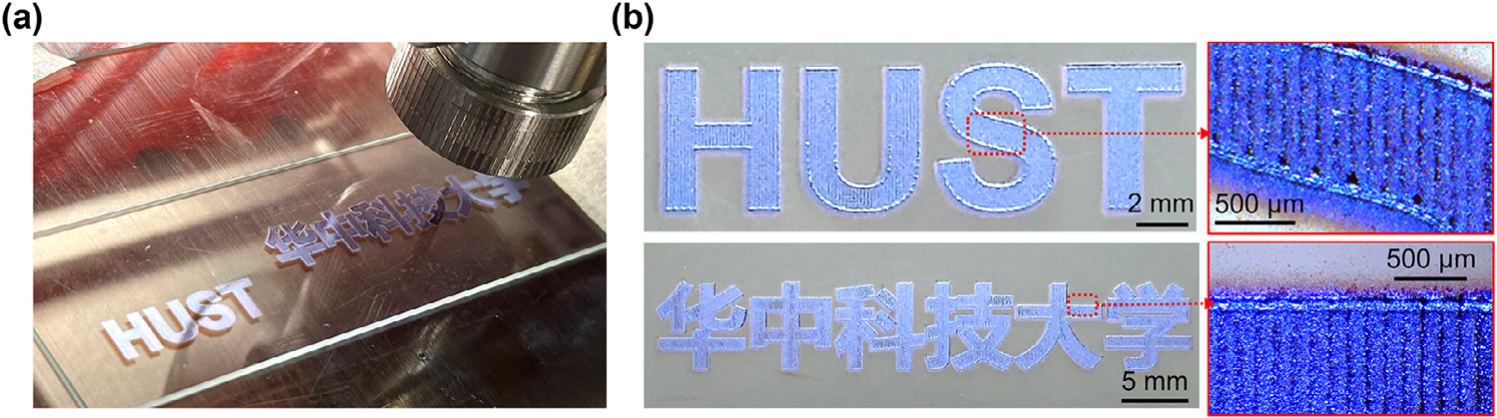
Complex patterns printed by the AJP system. a) Photograph showing the printing process. b) Overall and locally‐magnified images of two patterns.

To demonstrate the capability of the AJP system for manufacturing complex patterns, two distinct patterns (the abbreviation and full name of the authors affiliation, Huazhong University of Science and Technology) were designed and printed. During the printing, parallel lines with a hatch spacing of 127 µm was first employed to infill the patterns, and contour tracing was then applied along the outlines. The printing durations for these patterns were ≈6 and 15 min, respectively. As shown in **Figure** **11**, the printed patterns had accurately replicated the original design, which showed well‐defined edges without any protrusions or depressions. Minor ink splatter observed along the pattern outlines was attributed to the intentionally configured elevated gas flow rates and small focus ratio, which were implemented to shorten the printing time. The test result shows that the pattern has excellent electrical conductivity after sintering (Figure S3, Supporting Information).

**Figure 12:**
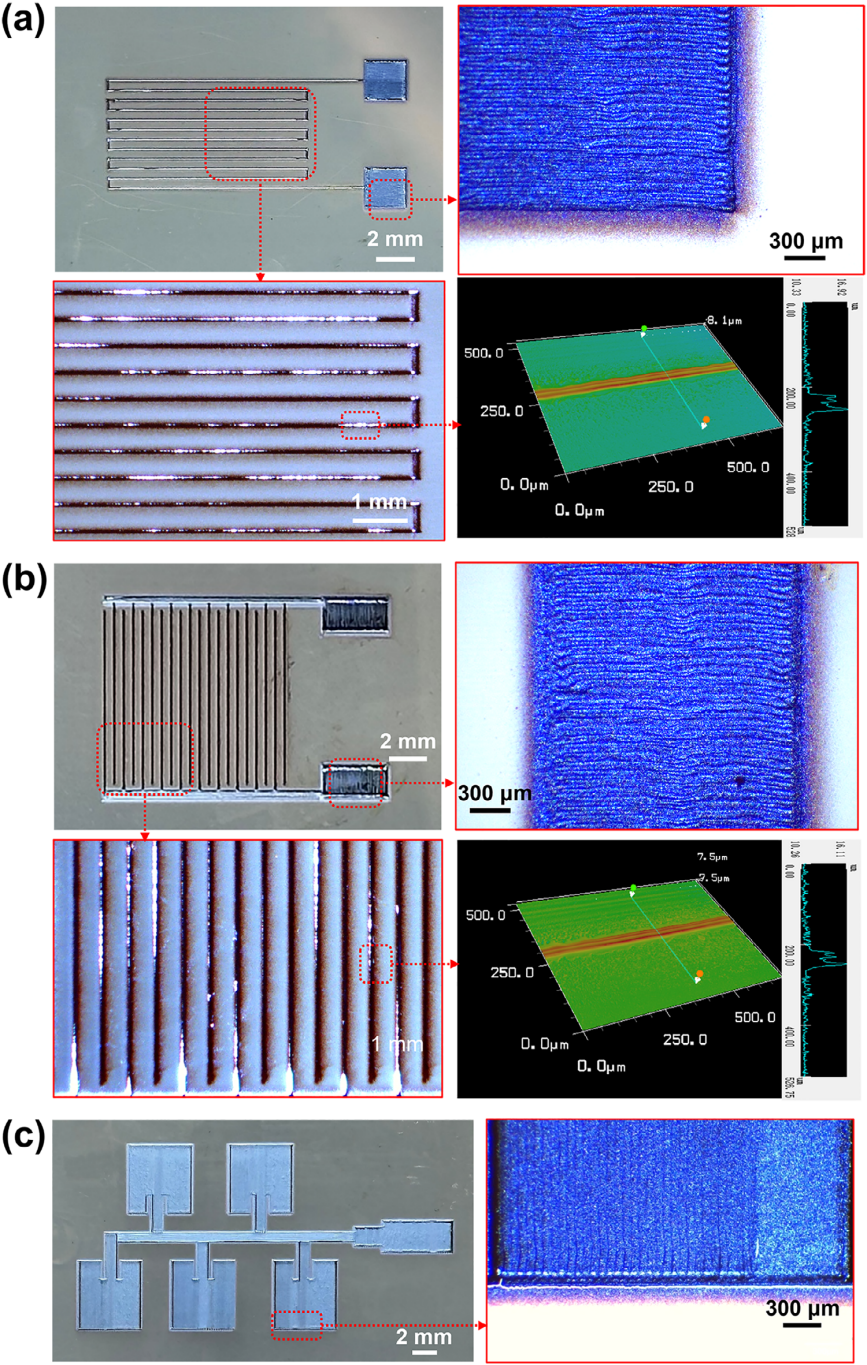
Images of three electronic devices printed by the AJP system. a) Temperature sensor. b) Interdigital capacitor. c) Microstrip array antenna.

Furthermore, three kinds of electrically functional devices were printed via AJP, as shown in **Figure** **12**, including a temperature sensor in Figure 12a, an interdigital capacitor in Figure 12b, and a microstrip array antenna in Figure 12c. All printings employed parallel‐line infill with a hatch spacing of 31 µm. The respective printing durations were ≈4 min, 4 min, and 1 h, corresponding to 155, 272, and 1696 times of ON‐OFF switching. The magnified micrographs reveal that all the printing traces showed uniform morphologies and well‐defined edges. Specially, the pattern displayed in Figure 12c was printed via as high as 1696 times ON‐OFF switching, where the overlapping parallel lines maintained well‐defined edges. These results confirm the capability of AJP system to achieve large‐format patterns manufacturing with high‐ responsivity and stable ON‐OFF jet control.

## 3 Conclusion

In this work, a high‐responsivity shuttering scheme to fundamentally eliminate the jet relaxation phenomenon based on keeping the internal pressure within printhead constant during the ON‐OFF switching process was proposed by a switching valve‐assisted AJP system. This breakthrough yields the significant enhancement in dimensional accuracy of complex patterns printed by AJP. The conclusions are summarized as follows:

1. The pressure within printhead was significantly affected by the OFF‐channel′s internal diameter in mechanical switching valve, including the outlet inner diameter *D*_2_ and the middle inner diameter *D_0_*. When *D_0_* = 2 mm and *D*_2_ = 0.5 mm, the pressure within printhead could be maintained virtually unchanged during the valve ON‐OFF switching cycles, making the jet relaxation time *T* eliminated within measurement uncertainty, which was more than 35 s during traditional AJP processes. Moreover, the jet relaxation elimination effect was independent of variations in printing parameters.
2. The ON‐OFF delay characteristics of the mechanical switching valve assisted AJP system was investigated. The jetting dead zone and switch dead zone within switching valve caused an ON‐OFF delay of ≈50 ms, resulting in an accuracy error Δ*L* of around 100 µm between the endpoints of opposite‐direction printed lines. Specifically, a displacement compensation was introduced in this research, making above ON‐OFF delay eliminated completely.
3. Short‐line arrays with different interval were prepared by varying the valve ON‐OFF frequency *F* from 0.2 to 50 Hz. Results indicate that the jet relaxation and ON‐OFF delay were all reduced below the detection limit (< 5 ms), demonstrating that the AJP system proposed in this paper had maintained the accurate jet control at *F* = 50 Hz, and it showed stable transient response characteristics. Meanwhile, the versatility of our shuttering scheme across different material types was also validated.
4. A number of large‐format patterns and functional devices were printed by AJP. The well‐defined edges without any protrusions or indentations were observed in all patterns, confirming the capability of AJP system to achieve large‐format patterns manufacturing with high‐response and stable ON‐OFF jet control.

On the basis of the mechanical switching valve assisted AJP system in this paper, we will focus on improving the printing efficiency in future, facilitating the manufacture of conformal devices on curved substrates.

## 4 Experimental Section

### Ink and Substrate

Two kinds of nano‐silver inks and one particle‐free silver ink were used. Both the nano‐silver inks were composed of silver nanoparticles (with particles size less than 10 nm, Figure S1, Supporting Information) as functional phase, N‐hexane as non‐polar organic solvent, and isopropanol as dispersant. The contents of silver nanoparticles therein were 40 and 60 wt.%. respectively, corresponding to the viscosities of inks ≈10 and ≈30 cp. Particle‐free silver ink was synthesized by reacting a silver nitrate (AgNO_3_) solution with a sodium citrate (Na_3_C_6_H_5_O_7_) solution, followed by drying the product, dispersing it in ethanol, finally adding sec‐butylamine (C_4_H_11_N) as a complexing agent. The viscosity of particle‐free silver ink was ≈117 cp. Microscope slides (Sail brand, China) was used as printing substrate.

### Equipment

A self‐made AJP system was conducted, it was mainly composed of four parts: atomizer, virtual impactor, printhead and mechanical switching valve embedded in the printhead. The AJP system adopted a pneumatic atomizer with argon as the carrier and sheath gas. The electronic ink was atomized into aerosol droplets by carrier gas (*Q*_A_) and subsequently flowed into the virtual impactor, where droplet size filtration and concentration enhancement were achieved through precisely adjusting the exhaust gas flow rate (*Q*_E_). Subsequently, the aerosol stream of *Q*_M_ (*Q*_M_ = *Q*_A_−*Q*_E_) was delivered into the printhead and formed a collimated aerosol jet stream surrounded by sheath gas of *Q*_Sh_, which passed through the nozzle and deposited on substrate to form the designed patterns. The *Q*_E_ was set to 0 sccm in this work. The AJP system was also integrated with a three‐axis motion stage, a charge coupled device (CCD) camera, three gas mass flow controllers (MFCs) (Siargo Ltd.) and two pneumatic pressure sensors (PSE543, SMC, Japan), a data acquisition card (USB‐6009, National Instrument, USA) to realize the printing of functional patterns and the monitor of internal pressure change during printing. The physical photograph of the switching valve assisted AJP system is shown in Figure S2 (Supporting Information). The printing nozzle (Musashi Inc., Japan) with inner diameter of 500 µm was adopted, the stand‐off distance was set to 2 mm and the printing speed was 2 mm s^−1^.

### Method

Drawing the design patterns with AutoCAD software; Converting the “.dwg” files to “.grb” path files, and importing it in a self‐developed printing software; Setting the flow rates of carrier gas *Q*_A_ and sheath gas *Q*_Sh_ by adjusting MFCs; Starting the printing process after the flow rate of aerosol jet stream outside nozzle was stabilized, by which the patterning samples were obtained; Drying the samples at 80 °C for 5 min; To test the conductivity, sintering the samples at 250 °C for 30 min.

### Measurements

The surface morphologies and 3D topographies of printed patterns were observed by stereomicroscope (SMZ171, Motic) and laser confocal microscopy (VK‐X200K, KEYENCE), respectively.

## Conflict of Interest

The authors declare no conflict of interest.

## Supporting information



Supporting Information

## Data Availability

The data that support the findings of this study are available from the corresponding author upon reasonable request.

## References

[1] S. M. Duan , X. C. Ren , X. T. Zhang , S. S. Cheng , W. P. Hu , Progr. Chem. 2018, 30, 429.

[2] C. Rosenbaum , M. Murphy , P. T. Lawrence , C. Sirkoch , S. R. Schneeberg , K. Zigner , S. Morris , E. Richman , C. Anyanwu , E. Will , C. Wheeler , E. Reed , C. N. LaFratta , Int. J. Extrem. Manuf. 2022, 4, 035001.

[3] K. K. B. Hon , L. Li , I. M. Hutchings , CIRP Ann. 2008, 57, 601.

[4] Q. Y. Zheng , B. Xie , Z. L. Xu , H. Wu , Int. J. Extrem. Manuf. 2023, 5, 035002.

[5] H. Yi , X. Q. Guo , F. L. Chang , H. J. Cao , J. An , C. K. Chua , Int. J. Extrem. Manuf. 2025, 7, 032002.

[6] Y. Li , G. Zhang , J. Zhang , D. Song , C. Guo , W. Zhou , Z. Fu , X. Zhu , F. Wang , Y. Duan , J. Dong , H. Lan , Int. J. Extrem. Manuf. 2025, 7, 012008.

[7] J. M. Hoey , A. Lutfurakhmanov , D. L. Schulz , I. S. Akhatov , J. Nanotechnol. 2012, 2012, 324380.

[8] N. J. Wilkinson , M. A. A. Smith , R. W. Kay , R. A. Harris , Int. J. Adv. Manuf. Technol. 2019, 105, 4599.

[9] E. B. Secor , Flex. Print. Electron. 2018, 3, 035002.

[10] M. Smith , Y. S. Choi , C. Boughey , S. Kar‐Narayan , Flex. Print. Electron. 2017, 2, 015004.

[11] Y.‐G. Park , I. Yun , W. G. Chung , W. Park , D. H. Lee , J.‐U. Park , Adv. Sci. 2022, 9, 2104623. 10.1002/advs.202104623 PMC8922115 35038249

[12] X. Konstantinou , X. Lan , K. Nguyen , A. Escorcia , R. Sandhu , J. Tice , IEEE Microw. Wirel. Technol. Lett. 2023, 33, 511.

[13] P. Li , J. Fleischer , E. Quinn , D. Park , J. Manuf. Mater. Process. 2024, 8, 39.

[14] L. Gamba , J. A. Lajoie , T. R. Sippel , E. B. Secor , Adv. Funct. Mater. 2023, 33, 2304060.

[15] S. Lu , J. A. Cardenas , R. Worsley , N. X. Williams , J. B. Andrews , C. Casiraghi , A. D. Franklin , ACS Nano 2019, 13, 11263. 31578857 10.1021/acsnano.9b04337

[16] K. Hong , Y. H. Kim , S. H. Kim , W. Xie , W. D. Xu , C. H. Kim , C. D. Frisbie , Adv. Mater. 2014, 26, 7032. 24975133 10.1002/adma.201401330

[17] M. T. Rahman , A. Rahimi , S. Gupta , R. Panat , Sens. Actuators A Phys. 2016, 248, 94.

[18] H. D. Liu , H. J. Zhang , W. Q. Han , H. J. Lin , R. Z. Li , W. Huang , Adv. Mater. 2021, 33, 2004782. 10.1002/adma.202004782 33448066

[19] P. Wang , C. L. Tang , Appl. Surf. Sci. 2024, 655, 159612.

[20] M. A. Ali , C. Hu , S. Jahan , B. Yuan , M. S. Saleh , E. Ju , S.‐J. Gao , R. Panat , Adv. Mater. 2021, 33, 2006647. 33349975 10.1002/adma.202006647 PMC7883076

[21] A. Mette , P. L. Richter , M. Horteis , S. W. Glunz , Prog. Photovolt. Res. Appl. 2007, 15, 621.

[22] C. Yang , E. Zhou , S. Miyanishi , K. Hashimoto , K. Tajima , ACS Appl. Mater. Interfaces 2011, 3, 4053. 21916457 10.1021/am200907k

[23] C. Hu , S. Jahan , B. Yuan , R. Panat , Adv. Sci. 2025, 12, 2405334. 10.1002/advs.202405334 PMC12005755 39921318

[24] N. T. H. Farr , M. Davies , J. Nohl , K. J. Abrams , J. Schäfer , Y. Lai , T. Gerling , N. Stehling , D. Mehta , J. Zhang , L. Mihaylova , J. R. Willmott , K. Black , C. Rodenburg , Adv. Sci. 2024, 11, 2306561. 10.1002/advs.202306561 PMC10933619 38145339

[25] S. Ramesh , Z. H. Xu , I. V. Rivero , D. R. Cormier , J. Manuf. Process. 2023, 95, 312.

[26] M. A. Mosa , J. Y. Jo , K.‐S. Kwon , J. Manuf. Process. 2024, 131, 694.

[27] T. Ma , Y. Li , H. Cheng , Y. Niu , Z. Xiong , A. Li , X. Jiang , D. Park , K. Zhang , C. Yi , Nat. Commun. 2024, 15, 6317. 39060314 10.1038/s41467-024-50789-w PMC11282100

[28] S. M. Ritchie , S. Kovacevic , P. Deshmukh , A. D. Christodoulides , J. A. Malen , S. D. Mesarovic , R. P. Panat , Nat. Commun. 2023, 14, 2667. 37160902 10.1038/s41467-023-38142-z PMC10169797

[29] S. M. Ritchie , C. S. Hu , R. Panat , Addit. Manuf. 2024, 95, 104549.

[30] S. Jahan , C. S. Hu , B. Yuan , S. M. Ritchie , R. Panat , Addit. Manuf. 2024, 92, 104385.

[31] M. Zeng , Y. Du , Q. Jiang , N. Kempf , C. Wei , M. V. Bimrose , A. N. M. Tanvir , H. Xu , J. Chen , D. J. Kirsch , J. Martin , B. C. Wyatt , T. Hayashi , M. Saeidi‐Javash , H. Sakaue , B. Anasori , L. Jin , M. D. McMurtrey , Y. Zhang , Nature 2023, 617, 292. 37165239 10.1038/s41586-023-05898-9 PMC10172128

[32] B. I. Guyll , L. D. Petersen , C. L. Pint , E. B. Secor , Adv. Funct. Mater. 2024, 34, 2316426.

[33] I. Sieber , D. Zeltner , M. Ungerer , A. Wenka , T. Walter , U. Gengenbach , Aerosol Sci. Technol. 2022, 56, 355.

[34] H. N. Zhang , J. Y. Huang , X. G. Zhang , C. N. Wong , Adv. Eng. Inform. 2025, 65, 103175.

[35] S. Ramesh , C. Mahajan , S. Gerdes , A. Gaikwad , P. Rao , D. R. Cormier , I. V. Rivero , Addit. Manuf. 2022, 59, 103090.

[36] R. R. Tafoya , E. B. Secor , Flex. Print. Electron. 2020, 5, 015009.

[37] A. Mahajan , C. D. Frisbie , L. F. Francis , ACS Appl. Mater. Interfaces. 2020, 5, 4856. 10.1021/am400606y 23659570

[38] J. D. Rurup , E. B. Secor , J. Manuf. Process. 2024, 120, 1231.

[39] G. Chen , Y. Gu , H. Tsang , D. R. Hines , S. Das , Adv. Eng. Mater. 2018, 20, 1701084.

[40] I. Grunwald , E. Groth , I. Wirth , J. L. Schumacher , M. Maiwald , V. Zoellmer , M. Busse , Biofabrication 2010, 2, 014106. 20811121 10.1088/1758-5082/2/1/014106

[41] M. Essien , D. M. Keicher , US10086622B2 2017, https://www.patents.google.com/patent/US10086622B2/en.

[42] K. K. Christenson , M. J. Renn , J. A. Paulsen , J. H. Hamre , C. Conroy , J. Q. Feng , Optomec Inc, US10632746 B2 2019, https://www.patents.google.com/patent/US10632746B2/en.

[43] J. S. Wright , C. M. Conroy , K. K. Christenson , J. D. Hamre , Optomec Inc, US12172444 B2 2022, https://www.patents.google.com/patent/US12172444B2/en.

[44] M. A. Mosa , J. Y. Jo , K.‐S. Kwon , Addit. Manuf. 2023, 67, 103466.

[45] M. A. Mosa , J. Y. Jo , S.‐H. Park , K.‐S. Kwon , Small 2025, 21, 2504037. 40470602 10.1002/smll.202504037 PMC12332830

[46] D. S. Miller , Internal Flow: A Guide to Losses in Pipe and Duct Systems, British Hydromechanics Research Association, Shanghai Library, Cranfield 1971.

