# Supplementary material for: Enhanced Responsivity, Accuracy, and Stability of Aerosol Jet Printing via Mechanical Switching Valve Assisted Internal Shuttering
Source: Adv Sci (Weinh). 2025 Nov 29;13(9):e19959. doi: 10.1002/advs.202519959 (PMC12903974; doi:10.1002/advs.202519959)
Supplement: Supplementary file 1 — Supporting Information [file ADVS-13-e19959-s001.docx]

Supporting Information

Enhanced Responsivity, Accuracy and Stability of Aerosol Jet Printing via Mechanical Switching Valve Assisted Internal Shuttering

*Jinhang Wang* ^1^*, Li Meng* ^1,^ **, Shuhuan Zhang* ^1^*, Pan Chen* ^1^*, Kaiwen Wei* ^1^*, Beibei Zhu* ^1^*, Xiaoyan Zeng* ^1^

^1^ Wuhan National Laboratory for Optoelectronics, School of Optical and Electronic Information, Huazhong University of Science and Technology, Wuhan, Hubei 430074, China.

**The silver-ink morphology**


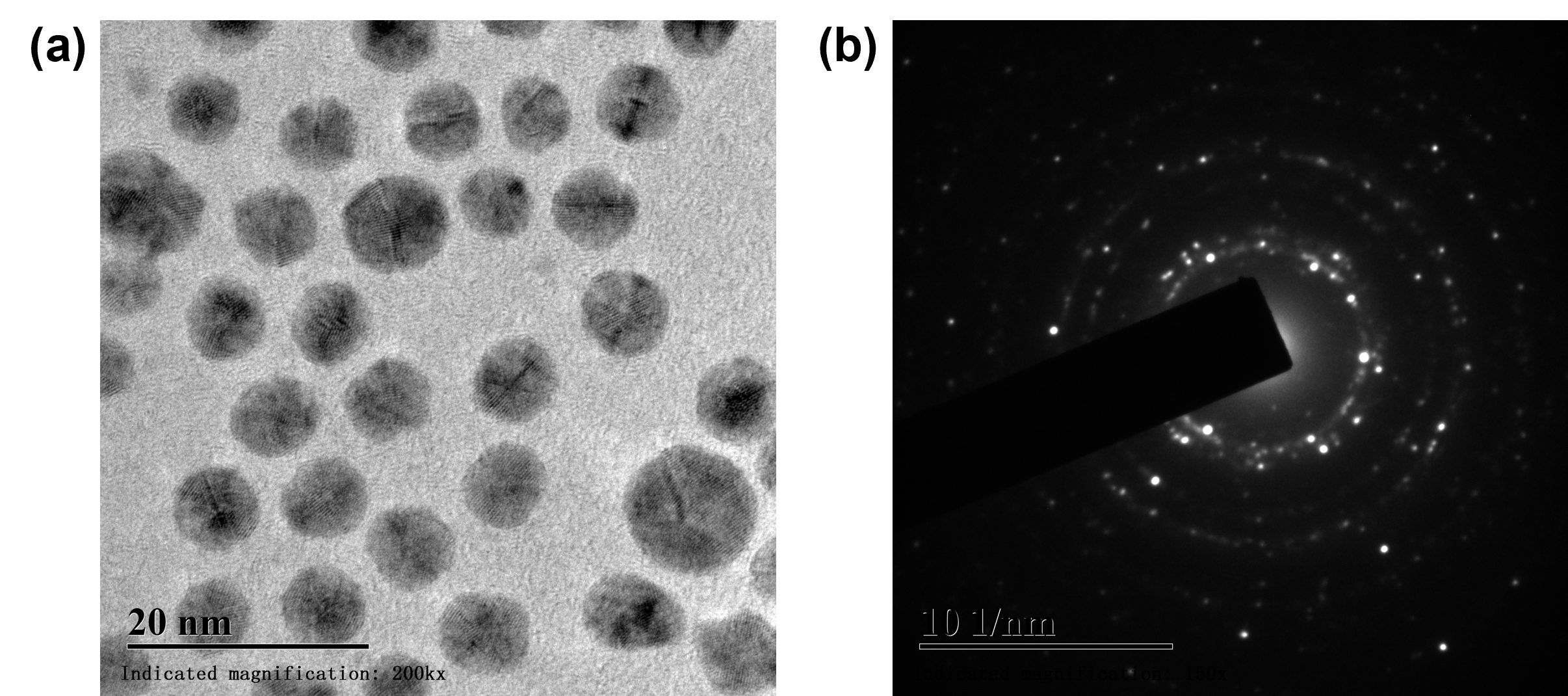


Figure S1. (a) Micromorphology and (b) a selected area electron diffraction (SAED) pattern of silver nanoparticles in the ink.

**The photograph of AJP system used for experiments**


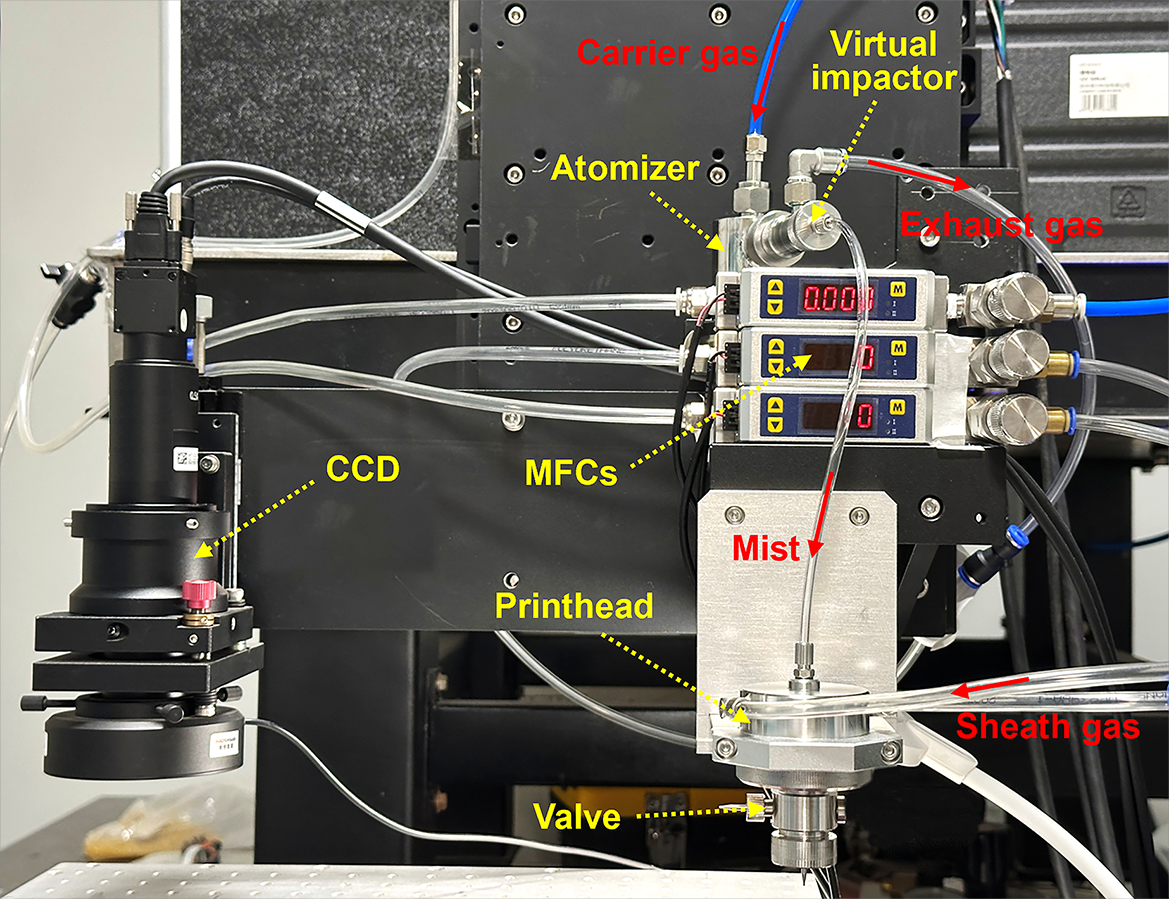


Figure S2. Photograph of the AJP system with an internal mechanical switching valve.

**Conductivity test of printed pattern**


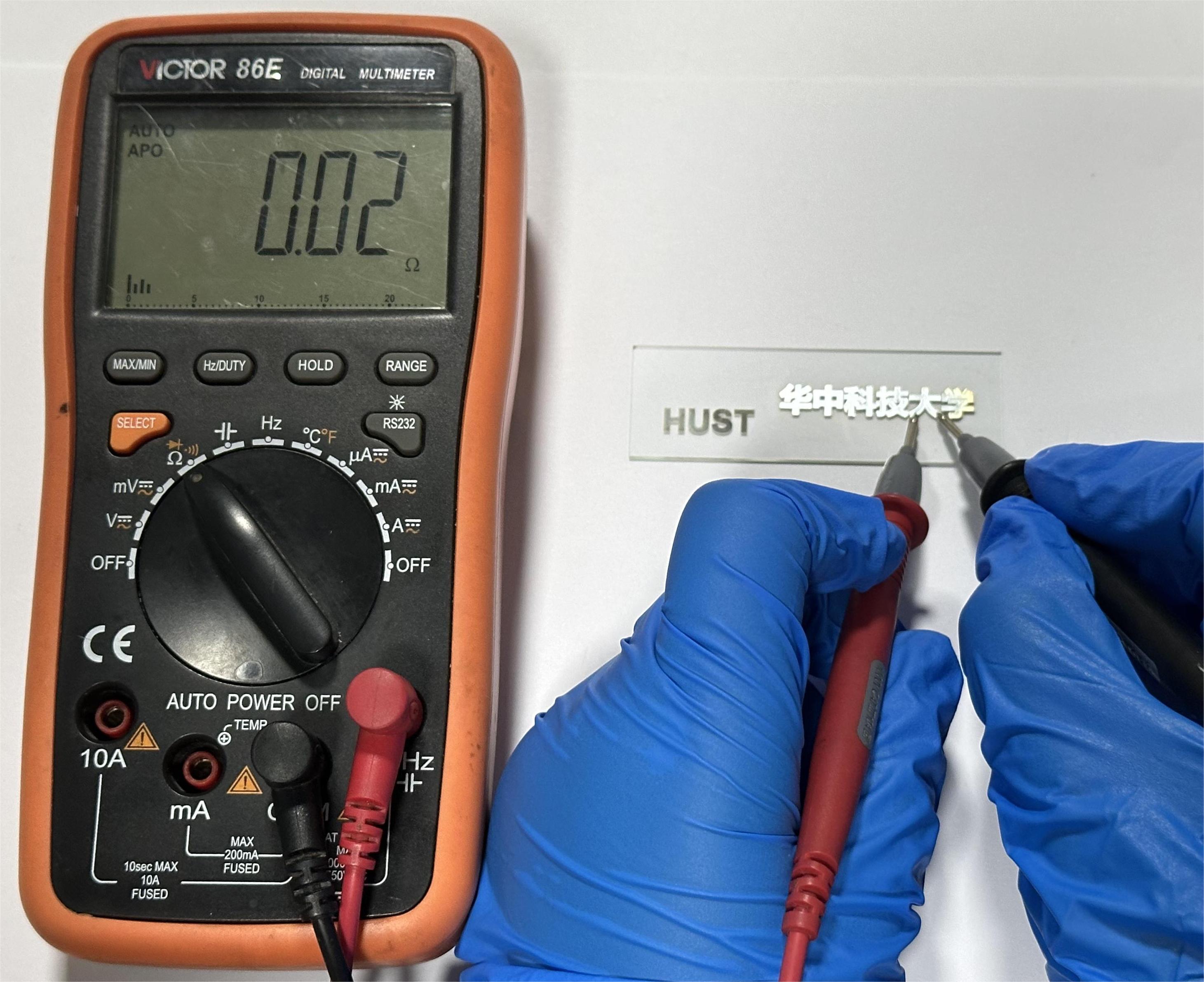


Figure S3. Conductivity test of printed pattern sintered at 250 ℃ for 30 min.
